# Factors influencing inferior alveolar nerve injury after extraction of mandibular third molar

**DOI:** 10.4317/medoral.26576

**Published:** 2024-08-01

**Authors:** Haijun He, Niansu Ruan

**Affiliations:** 1 Department of Stomatology, Wuhan No.1 Hospital (Wuhan Hospital of Traditional Chinese and Western Medicine), Wuhan, Hubei, China; 2 State Key Laboratory of Oral and Maxillofacial Reconstruction and Regeneration, Key Laboratory of Oral Biomedicine Ministry of Education, Hubei Key Laboratory of Stomatology, School and Hospital of Stomatology, Wuhan University, Hubei, China

## Abstract

**Background:**

This study sought toexplore the manifestations of clinical symptoms and identify the risk factors linked to inferior alveolar nerve injury (IANI) in the context of mandibular third molar extraction.

**Material and Methods:**

In this study, 172 patients admitted to our hospital for mandibular third molar extraction from June 2021 to December 2022 were selected for the study, and the clinical data of the participants were retrospectively analyzed, and the risk factors of IANI associated with mandibular third molar extraction were analyzed by uni/multi-factor logisitic regression.

**Results:**

Noticeable distinctions were noted between the groups with and without injuries in relation to age, time of surgery, number of broken roots, angle of blockage, CEJ (cementoenamel junction) distance, curved roots of the molar, clarity of the upper and lower walls of the nerve canal, and Pell & Gregory classification. Logistic regression analysis showed that age, time of surgery, number of broken roots, angle of blockage (40°-70°), CEJ distance (10-12 mm, >12 mm), and curved roots of the molar were independent risk factors for IANI. Multi-factor logistic regression analysis further confirmed that age, number of broken roots, angle of blockage (40°-70°), CEJ distance (10-12 mm, >12 mm), and curved roots of the molar were associated with IANI.

**Conclusions:**

Alveolar nerve injury manifests as hyperalgesia or absence of sensation, numbness and abnormal pain in the lower lip. Factors influencing IANI associated with mandibular third molar extraction were age, number of broken roots, angle of blockage (40°-70°), CEJ distance (10-12 mm, >12 mm), and curved roots of the molar.

** Key words:**Mandibular third molar, extraction, inferior alveolar, nerve injury, influencing factors.

## Introduction

The third mandibular molar, commonly known as the wisdom tooth, frequently encounters obstructive issues that prevent it from erupting into its normal functional position. This can be attributed to a variety of factors, including limited jaw space and resistance from neighboring teeth during the eruption process. The incidence of such obstructive cases ranges from 22% to 54% (1). Mandibular third molar obstruction often causes pericoronitis, caries, extra-root resorption, crowding, odontogenic cysts and tumors in wisdom teeth. However, its anatomical location makes surgery more difficult due to poor visualization. Various postoperative complications such as bleeding, infection, edema, dry socket, mouth opening restriction, and nerve injury may arise (2,3). In addition, the inferior alveolar nerve emanates from the mandibular branch of the trigeminal nerve. It primarily provides sensory innervation for the pulp, periodontium, alveolar bone, and lower front teeth of the mandible, transmitting pain and tactile sensations (3). During extraction of the third mandibular molar, the inferior alveolar nerve may be damaged directly by surgical instruments or indirectly by root displacement and compression, which may cause dull or abnormal sensation in the patient's lower lip, chin, and gums (4). Injury to the inferior alveolar nerve stands out as a prevalent and significant complication following the extraction of the mandibular third molar, exhibiting an incidence rate of0.4%-8.4% and permanent injury of 0.014%-3.6%, and the likelihood of inferior alveolar nerve injury (IANI) is as high as 10%-35% in high-risk populations (1,5). Injury to the inferior alveolar nerve results in temporary or permanent loss of sensation in its primary innervation area. Symptoms such as numbness of the affected lower lip, chin and gums of the lower jaw are often seen. In severe cases, it affects the patient's speech, diet and other daily activities, potentially impacting the individual's well-being, both in terms of physical and mental health, and overall quality of life (6). Due to its low incidence and predicTable improvement in neurological symptoms, clinicians have paid little attention to inferior alveolar nerve injuries. However, preoperative evaluation and prevention of IANI is still necessary.

Numerous risk factors for IANI have been identified by scholars worldwide, including age, depth of anesthesia, operator expertise, and intraoperative exposure of the nerve canal. Among these factors, the distance and positioning of the root in relation to the inferior alveolar nerve canal are considered the most critical (7). However, no uniform conclusions have been reached regarding the risk factors of the inferior alveolar nerve canal. Furthermore, there is a scarcity of clinical investigations focusing on the symptoms associated with IANI following the extraction of mandibular third molars. Thus, this study will investigate the clinical symptoms and influencing factors of IANI associated with mandibular third molar extraction.

## Material and Methods

- Clinical data

One hundred and seventy-two patients with mandibular third molars extracted in our hospital from June 2021 to January 2022 were categorized into the uninjured group (157 cases) and the injured group (15 cases) according to whether they had IANI; age: 22-34 years, mean age (29.97±5.01) years; gender: 74 males and 98 females. All procedures adhered to the applicable guidelines and regulations. Approval for the study was granted by the medical ethics committee at our institution, and all individuals provided their consent by signing an informed consent form after being briefed on the study's objectives. 

- Inclusion criteria

1 Inclusion of patients who have undergone lower third molar extraction, performed by skilled dental surgeons (8).

2 Participants must be over 18 years old.

3 Inclusion of patients with confirmed lower alveolar nerve injury based on pre- and post-operative imaging examinations such as X-rays, CT scans.

4 Presence of the lower second molar with normal functional ability in patients.

5 Consistent clinical symptoms of lower alveolar nerve injury must be present, such as numbness, tingling, reduced sensation, etc.

6 Patients with no significant underlying diseases, complications, and contraindications for tooth extraction, ensuring that the research results are not influenced by other factors.

- Exclusion criteria

1 Combined cysts, tumors or the presence of acute inflammation in the mandibular third molar;

2 Incomplete root development of the mandibular third molar;

3 Presence of preoperative sensory disturbance in the inferior alveolar innervation area;

4 Those who did not want to participate in this study.

- Surgery

The surgical interventions were consistently conducted by a single surgeon. The procedural sequence encompasses the subsequent steps:

Flap: A routine flap technique is employed to fully expose the crown of the affected tooth.

Debridement: The bone tissue covering the crown surface is completely removed to ensure full visualization of the crown.

Crown removal: The crown is carefully removed in a stepwise manner, taking different directions into consideration.

Root splitting: The location for root splitting is determined based on preoperative CBCT analysis. Multiple roots are separated from each other and transformed into single roots, malformed roots, or single hypertrophied roots. An appropriate amount of periapical debridement is performed, followed by gradual displacement of the roots for complete extraction.

Suture: After thorough cleaning of the extraction sockets, the mucosal flap is repositioned and aligned, and sutures are applied.

- Clinical Data Collection

Clinical data were collected from all the participants including age, gender, BMI, number of roots, mandibular canal morphology, direction of obstruction, time of surgery, number of broken roots, angle of blockage, CEJ distance, curved roots of molars, whether the upper and lower walls of the neural canal were clear and Pell & Gregory classification (9).

Measurement of the direction of interruption of the mandibular third molar: according to Winter's classification (10), the intersection angle between the long axis of the mandibular second molar and third molar was measured according to the panoramic film to an accuracy of -10°-10° for vertical obstruction, 11°-79° for submedially inclined obstruction, and 80°-100° for horizontal obstruction.

Angle measurement of mandibular third molar interruptions (11): The interceptive angle of the third molar was measured according to the SHILL-ER method, and the interceptive angle was the angle of intersection between the plane of the mandibular second molar and the plane of the mandibular third molar, measured to an accuracy of 1° according to the panoramic film. The interceptive angles were grouped: 0°~, 10°~, 40°~, and 70°~.

Mandibular second molar to third molar CEJ distance measurement: the mandibular second molar to third molar CEJ distance was measured according to the method of LEONE*et al*. (12). The CEJ distance was the distance between the distal mesial enamel bone boundary of the second molar and the proximal mesial enamel bone boundary of the third molar, measured to 1 mm based on panoramic films. the CEJ distances were grouped: 1 to 3 mm, 4 to 6 mm, 7 to 9 mm, 10~12 mm, and >12 mm.

Oral surface tomogram: A PlanmecaProMax oral surface tomograph (Finland) was used to take surface tomograms. The participants were placed in a standing position with the cervical spine vertical, with the chin in the middle of the chin rest, the incisive edge of the anterior teeth occluded in the plate socket, the sagittal plane of the head perpendicular to the ground, and the angular parallels of the orbito-ear line and the auditory-nasal line parallel to the ground. Scanning conditions: tube voltage 68 kV, tube current 10 mA, scanning time 16.6 s.

Reading: After the digital panoramic view was displayed on the computer using KinstaWeining digital software, the same physician performed the reading and analysis of the oral digital surface tomograms for data acquisition. The categorization of the inferior alveolar nerve canal involved three distinct classes based on its clarity in the apical region of IMTM: 1) both upper and lower walls were clear; 2) the upper wall was unclear and the lower wall was clear; 3) both upper and lower walls were unclear. To record whether each root of the IMTM (Impacted Mandibular Third Molar) to be extracted was curved or not. The IMTM was classified into three types according to Pell&Gregory classification: high obstruction, middle obstruction, and low obstruction.

- Statistical analysis 

The data analysis utilized SPSS 21.0 software, with Excel employed for database creation. Measurement data following a normal distribution were represented as mean±sd, and one-way ANOVA was used for overall comparison of the data in each group, and LSD was used for two-way comparison of the data between and within groups; the count data were expressed as rate (%), and the chi-square χ2 test was used for comparison; one-/multi-factor Logisitic regression was used to analyze the influence factors, and *p*<0.05 was considered as significant difference.

## Results

- Comparison of clinical symptoms between the two groups

The rate of IANI after extraction of mandibular third molar in this study was 8.72% (15/172). When comparing the age, gender, BMI, number of roots, mandibular canal morphology, and direction of blockage between the two groups, the difference was not significant (*p*> 0.05); Statistical significance (*p*< 0.05) was observed in the comparisons of the time of surgery, number of broken roots, angle of blockage, CEJ distance, curved roots of molars, whether the upper and lower walls of the nerve canal were clear, and Pell & Gregory classification between the two groups; the injury group presented with hypoesthesia or absence of sensation in the lower lip, numbness and abnormal pain. See Table 1.

- Univariatelogisitic regression analysis of factors influencing IANI associated with mandibular third molar extraction

The independent variables were defined as the data exhibiting variances in the comparative analysis of the above clinical data including age, time of surgery, number of broken roots, angle of blockage, CEJ distance, curved root of the molar, clarity of the upper and lower walls of the nerve canal, and Pell & Gregory classification, and the dependent variables were the influencing factors of IANI associated with mandibular third molar extraction, and univariatelogisitic regression analysis was performed. The influencing factors of IANI associated with mandibular third molar extraction were age, time of surgery, number of broken roots, angle of blockage (40°-70°), CEJ distance (10-12 mm, >12 mm), and curved roots of the molar, as shown in Table 2

- Multifactorial logisitic regression analysis of factors influencing IANI associated with extraction of mandibular third molars

The independent variables were set as the data with differences in the comparison of the above clinical data including age, time of surgery, number of broken roots, interceptive angle (40°-70°), CEJ distance (10-12 mm, >12 mm), and molar curved root, and the dependent variables were the influencing factors of IANI associated with mandibular third molar extraction, and multifactorial logisitic regression analysis was performed. The influencing factors for IANI associated with third molar extraction were age, number of broken roots, angle of interruption (40°-70°), CEJ distance (10-12 mm, >12 mm), and molar curved roots, as shown in Table 3.

## Discussion

The removal of the mandibular third molar stands out as a frequently performed surgical procedure within the realm of oral and maxillofacial surgery. Due to the special anatomical location, it is difficult for the clinician to directly observe the entire surgical area. When the clinician is inexperienced, it may cause damage to key structures such as nerves and blood vessels (13). The IANI may be directly damaged by surgical instruments or indirectly damaged by root displacement or compression during surgery (14). A study by Barry*et al*. (15) stated that the incidence of IANI after mandibular third molar extraction was 6.6%. Bataineh *et al*. (16) found that the incidence of postoperative IANI was 3.9%.The incidence of IANI after mandibular third molar extraction in our study was 8.72% (15/172), which is similar to the results of previous studies and is within a reasonable range. However, a study by Daware*et al*. (17) noted that the incidence of nerve injury after surgical removal of the mandibular third molar was 2%, which was significantly lower than in the present study, which may have been caused by the different study population included. In addition, IANI after mandibular third molar extraction decreases the quality of life of patients, so clinicians should avoid the occurrence of IANI after tooth extraction as much as possible.

The subjective symptoms of nerve injury are mainly described by the patient. Patients with IANI may present with a variety of symptoms, mainly including decreased or absent sensation of the lower lip, numbness and abnormal pain; lingual nerve injury mainly presents with persistent numbness and taste disturbance of the ipsilateral anterior 2/8 of the tongue, and a few have painful symptoms (18). Objective clinical tests for nerve injury include superficial nociception, tactile sensation, two-point discrimination sensation, deep nociceptive temperature sensation, and solid sensation. Electrophysiological diagnostic tests are done when necessary. If nerve injury symptoms do not ameliorate after 2 years, it is considered a permanent loss of function (19). The results of our study showed that the clinical characteristics of IANI after mandibular third molar extraction included the time of surgery, the number of broken roots, the angle of blockage, the CEJ distance, the curved roots of the molar, the clarity of the upper and lower walls of the nerve canal, and the Pell & Gregory classification. When the apical development is completed, the interrupted mandibular third molar has various root morphological variants and constitutes various complex relationships with the inferior alveolar nerve, and the extraction process is more likely to lead to nerve damage (20). Prophylactic extraction of mandibular interrupted third molars is generally recommended below the age of 25 years, and the ideal age is 16-22 years, at which the roots form 1/3 to 2/3. With age, various variations of the roots are frequent, and the percentage of inorganic components of human bone gradually increases, as well as hardness and elasticity, and the periodontal space becomes progressively narrower, making tooth extraction significantly more difficult and increasing the chance of IANI.Jerjes*et al*. (21)also confirmed that patients aged 26 and older had a higher incidence of IANI after surgery. Deeper obstruction increases the difficulty of extracting the mandibular third molar by making the tooth less visible and increasing the likelihood of a complex root tip relationship with the mandibular canal. This, in turn, raises the risk of IANI (22). A study by Benediktsdóttir*et al*. (23) suggested that both the root morphology and the degree of curvature affect the operative time. A study by Blondeau*et al*. (24) stated that the Pell&Gregory classification can be used as an influential factor in assessing nerve injury after mandibular third molar extraction. Guillaumet-Claure*et al*. (25) stated that the influential factors for nerve injury after mandibular third molar extraction were high, medium and low of Pell&Gregory. Rafiq*et al*.'s (26) study concluded that age and longer operative time were risk factors for IANI after mandibular third molar extraction. In Jin*et al*.'s (27) study pointed out that number of roots and depth of embedding were risk factors for the occurrence of IANI. Age was noted as an influencing factor for IANI associated with mandibular third molar extraction in a study by Tojyo*et al*. (28The results of our study showed that the factors influencing IANI associated with mandibular third molar extraction were operative time, number of broken roots, angle of interruption (40°-70°), CEJ distance (10-12 mm, >12 mm), and molar bending roots, which were largely consistent with the results of the above study.

This study has some limitations. Firstly, it is a retrospective study, and surgical-related variables such as surgical difficulty and intraoperative exposure of the inferior alveolar nerve canal were not included in the discussion. Additionally, the experience of different surgeons may have influenced the results (16,21,29Furthermore, other factors such as anesthesia modalities also need to be considered, as they can affect the incidence of IANI (30). Lastly, the sample size in this study was relatively small. Future studies should aim to expand the sample size or adopt multicenter clinical trials, and include more variables for investigation.

## Conclusions

In summary, lower alveolar nerve injury associated with mandibular third molar extraction manifested as decreased or absent lower lip sensation, numbness, and abnormal pain, and the factors influencing the injury were age, number of broken roots, angle of interruption (40°-70°), CEJ distance (10-12 mm, >12 mm), and curved roots of the molar.

## Figures and Tables

**Table 1:** Comparison of clinical data between the alveolar nerve injured and uninjured groups.

Variables		uninjured (n=157)	injured (n=15)	χ^2^/t	*p*
Age (years)		25.01±7.01	31.87±6.92	-3.625	<0.001
Sex (male)		71	3	3.705	0.054
BMI (kg/m2)		22.91±3.02	23.03±3.28	-0.146	0.884
Number of tooth roots	single root	113	11	0.013	0.911
	Multiple heel	44	4		
	Surgery time	10.61±1.28	13.81±1.32	-9.227	<0.001
Whether the number of broken roots (pcs)	Yes	2	5	38.835	<0.001
	No	155	9		
Mandibular canal morphology	Oval	42	4	0.033	0.983
	Dumbbell-shaped	66	6		
	Teardrop shape	49	5		
Direction of obstruction	Vertical obstruction	23	3	0.653	0.721
	Sub-mid inclination	110	9		
	Horizontal obstruction	24	3		
Obstruction angle	0°	19	1	4.911	0.178
	10°	28	2		
	40°	50	9		
	70°	60	3		
CEJ distance	1-3mm	9	1	3.914	0.418
	4-6mm	28	1		
	7-9mm	57	9		
	10-12mm	38	3		
	>12mm	25	1		
Blocking side	Right side	81	5	1.826	0.177
	Left side	76	10		
Whether the molar is curved root	Yes	115	2	22.597	<0.001
	No	42	13		
Is the upper and lower wall of the neural canal clear?	Both upper and lower are clear	33	12	25.758	<0.001
	The upper wall is not clear, the lower wall is clear	117	2		
	Upper and lower walls are not clear	7	1		
Pell and Gregory classification	High	25	7	8.581	0.014
	Middle	94	6		
	Low	38	2		
Hypoesthesia of the lower lip		-	6	-	-
Numbness of the lower lip		-	8	-	-
Abnormal pain in the lower lip		-	7	-	-

CEJ, cementoenamel junction

**Table 2:** Univariate logistic regression analysis of factors influencing inferior alveolar nerve injury associated with extraction of mandibular third molars.

Variables		β	SE	Wdlodχ^2^	OR(95%CI)	*p*
Age		0.132	0.037	17.632	1.137 (1.037-1.276)	<0.001
Time of surgery		1.537	0.491	10.579	4.321 (1.838-10.231)	0.001
Number of root breaks		1.428	0.462	10.271	4.081 (1.563-7.321)	<0.001
Obstruction angle	0°-10°	0.353	0.239	2.007	1.321 (0.856-2.098)	0.163
	40°-70°	0.281	0.137	4.387	1.326 (1.017-1.675)	0.031
CEJ distance	1-3mm	0.379	0.302	1.571	1.473 (0.781-2.391)	0.198
	4-6mm	0.089	0.116	0.683	1.091 (0.871-1.354)	0.402
	7-9mm	0.008	0.002	1.761	0.008 (0.991-1.014)	0.173
	10-12mm	0.792	0.301	6.781	2.216 (1.217-3.981)	0.007
	>12mm	0.653	0.289	4.651	1.927 (1.057-3.281)	0.028
Molar curved root		0.319	0.148	4.287	1.281 (1.022-1.763)	0.028
Is the upper and lower wall of the neural canal clear	Upper and lower are clear	0.143	1.049	0.021	1.154 (0.142-3.763)	0.836
	The upper wall is not clear, the lower wall is clear	0.348	0.683	0.218	1.387 (0.356-4.918)	0.643
	Upper and lower walls are not clear	0.072	0.267	0.048	1.065 (0.621-1.817)	0.832
Pell and Gregory classification	High	0.432	0.309	1.173	1.519 (0.821-2.748)	0.173
	Middle	0.378	0.297	1.876	1.498 (0.819-2.313)	0.165
	Low	0.193	0.173	1.093	1.303 (0.94-1.384)	0.539

CEJ, cementoenamel junction.

**Table 3:** Multifactorial logisitic regression analysis of factors influencing inferior alveolar nerve injury associated with extraction of mandibular third molars.

Variables		β	SE	Wdlodχ^2^	OR(95%CI)	*p*
Age		1.231	0.043	32.187	3.198 (3.091-3.276)	<0.001
Time of surgery		0.102	0.105	1.009	1.109 (0.872-1.432)	0.309
Number of severed roots		1.416	0.246	26.198	3.281 (1.298-4.198)	<0.001
Obstruction angle (40°-70°)		0.738	0.031	42.091	2.091 (1.761-2.321)	<0.001
CEJ distance	10-12mm	1.367	0.682	3.761	3.813 (1.049-13.291)	0.041
	>12mm	1.781	0.738	6.218	4.198 (1.281-18.981)	0.009
Molar curved root		0.827	0.379	4.981	2.287 (1.093-3.198)	0.023

CEJ, cementoenamel junction.

## Data Availability

The data that support the findings of this study are available from the Correspondence upon reasonable request.

## References

[1] Patel PS, Shah JS, Dudhia BB, Butala PB, Jani YV, Macwan RS (2020). Comparison of panoramic radiographand cone beam computed tomography findings for impacted mandibular third molar root and inferior alveolar nerve canal relation. Indian J Dent Res.

[2] Rivera-Herrera RS, Esparza-Villalpando V, Bermeo-Escalona JR, Martínez-Rider R, Pozos-Guillén A (2020). Agreement analysis of three mandibular third molar retention classifications. Gac Med Mex.

[3] Ye ZX, Qian WH, Wu YB, Yang C (2021). Pathologies associated with the mandibular third molar impaction. Sci Prog.

[4] Le Donne M, Jouan R, Bourlet J, Louvrier A, Ducret M, Sigaux N (2022). Inferior alveolar nerve allogenic repair following mandibulectomy: A systematic review. J Stomatol Oral Maxillofac Surg.

[5] Del Lhano NC, Ribeiro RA, Martins CC, Assis NMSP, Devito KL (2020). Panoramic versus CBCT used to reduce inferior alveolar nerve paresthesia after third molar extractions: a systematic review and meta-analysis. Dentomaxillofac Radiol.

[6] AlAli AM, AlAnzi TH (2021). Inferior alveolar nerve damage secondary to orthodontic treatment: a systematic scoping review. Int J Risk Saf Med.

[7] Ducic I, Yoon J (2019). Reconstructive options for inferior alveolar and lingual nerve injuries after dental and oral surgery: an evidence-based review. Ann Plast Surg.

[8] Almendros-Marqués N, Berini-Aytés L, Gay-Escoda C (2008). Evaluation of intraexaminer and interexaminer agreement on classifying lower third molars according to the systems of Pell and Gregory and of Winter. J Oral Maxillofac Surg.

[9] Gümrükçü Z, Balaban E, Karabağ M (2021). Is there a relationship between third-molar impaction types and the dimensional/angular measurement values of posterior mandible according to Pell & Gregory/Winter Classification?. Oral Radiol.

[10] Quek S, Tay C, Tay K, Toh S, Lim K (2003). Pattern of third molar impaction in a Singapore Chinese population: a retrospective radiographic survey. Int J Oral Maxillofac Surg.

[11] Shiller WR (1979). Positional changes in mesio-angular impacted mandibular third molars during a year. J Am Dent Assoc.

[12] Leone S, Edenfield M, Cohen M (1986). Correlation of acute pericoronitis and the position of the mandibular third molar. Oral Surg Oral Med Oral Pathol.

[13] Bao MZ, Liu W, Yu SR, Men Y, Han B, Li CJ (2021). Application of platelet-rich fibrin on mandibular third molar extraction: systematic review and Meta-analysis. Hua Xi Kou Qiang Yi Xue Za Zhi.

[14] Almadhoon HW, Hamdallah A, Eida MA, Al-Kafarna M, Atallah DA, AbuIriban RW (2022). Efficacy of different dexamethasone routes and doses in reducing the postoperative sequelae of impacted mandibular third-molar extraction: A network meta-analysis of randomized clinical trials. J Am Dent Assoc.

[15] Barry E, Ball R, Patel J, Obisesan O, Shah A, Manoharan A (2022). Retrospective evaluation of sensory neuropathies after extraction of mandibular third molars with confirmed "high-risk" features on cone beam computed topography scans. Oral Surg Oral Med Oral Pathol Oral Radiol.

[16] Bataineh AB (2001). Sensory nerve impairment following mandibular third molar surgery. J Oral Maxillofac Surg.

[17] Daware SN, Balakrishna R, Deogade SC, Ingole YS, Patil SM, Naitam DM (2021). Assessment of postoperative discomfort and nerve injuries after surgical removal of mandibular third molar: A prospective study. J Family Med Prim Care.

[18] Li Y, Ling Z, Zhang H, Xie H, Zhang P, Jiang H (2022). Association of the inferior alveolar nerve position and nerve injury: a systematic review and meta-analysis. Healthcare (Basel).

[19] Kasapoğlu MB, Doğancalı GE (2022). Inferior alveolar nerve injury due to the extrusion of calcium hydroxide during endodontic treatment: A case report. Aust Endod J.

[20] Sinha S, Duong T, Duy TD, Ko EC, Chen YR, Huang C (2022). Penetration of inferior alveolar nerve canal increased by bicortical fixation after bilateral sagittal split osteotomy in mandibular prognathism. Int J Oral Maxillofac Surg.

[21] Jerjes W, Upile T, Shah P, Nhembe F, Gudka D, Kafas P (2010). Risk factors associated with injury to the inferior alveolar and lingual nerves following third molar surgery-revisited. Oral Surg Oral Med Oral Pathol Oral Radiol Endod.

[22] Leung YY (2019). Management and prevention of third molar surgery-related trigeminal nerve injury: time for a rethink. J Korean Assoc Oral Maxillofac Surg.

[23] Benediktsdóttir IS, Wenzel A, Petersen JK, Hintze H (2004). Mandibular third molar removal: risk indicators for extended operation time, postoperative pain, and complications. Oral Surg Oral Med Oral Pathol Oral Radiol Endod.

[24] Blondeau F, Daniel NG (2007). Extraction of impacted mandibular third molars: postoperative complications and their risk factors. J Can Dent Assoc.

[25] Guillaumet-Claure MA, Juiz-Camps AM, Gay-Escoda C (2022). Prevalence of intraoperative and postoperative iatrogenic mandibular fractures after lower third molar extraction: A systematic review. J Clin Exp Dent.

[26] Rafiq A, Abbas I, Khan NS, Rafiq A (2023). Lingual Nerve Injury During Impacted Mandibular Third Molar Surgery. J Coll Physicians Surg Pak.

[27] Jin Q, Xie Z (2022). Evaluating the risk factors of inferior alveolar nerve injury following removal of the mandibular third molars. Zhonghua Kou Qiang Yi Xue Za Zhi.

[28] Tojyo I, Nakanishi T, Shintani Y, Okamoto K, Hiraishi Y, Fujita S (2019). Risk of lingual nerve injuries in removal of mandibular third molars: a retrospective case-control study. Maxillofac Plast Reconstr Surg.

[29] Jerjes W, Swinson B, Moles D, El-Maaytah M, Banu B, Upile T (2006). Permanent sensory nerve impairment following third molar surgery: a prospective study. Oral Surg Oral Med Oral Pathol Oral Radiol Endod.

[30] Brann C, Brickley M, Shepherd J (1999). Factors influencing nerve damage during lower third molar surgery. Br Dent J.

